# Lipid metabolic reprogramming of hepatic CD4^+^ T cells during SIV infection

**DOI:** 10.1128/spectrum.01687-23

**Published:** 2023-09-01

**Authors:** Julien A. Clain, Steven Boutrais, Juliette Dewatines, Gina Racine, Henintsoa Rabezanahary, Arnaud Droit, Ouafa Zghidi-Abouzid, Jérôme Estaquier

**Affiliations:** 1 Centre de Recherche du CHU de Québec, Université Laval, Québec City, Québec, Canada; 2 Proteomics Platform, CHU de Québec - Université Laval Research Center, Québec City, Québec, Canada; 3 Computational Biology Laboratory, CHU de Québec - Université Laval Research Center, Québec City, Québec, Canada; 4 INSERM U1124, Université Paris, Paris, France; National Institutes of Health, Bethsda, Maryland, USA

**Keywords:** AIDS, SIV, liver, CD4, metabolism, lipid, cytotoxic, granzyme, autophagy, ferroptosis, PD-1, CCR5, viral reservoir

## Abstract

While liver inflammation is associated with AIDS, little is known so far about hepatic CD4^+^ T cells. By using the simian immunodeficiency virus (SIV)-infected rhesus macaque (RM) model, we aimed to characterize CD4^+^ T cells. The phenotype of CD4^+^ T cells was assessed by flow cytometry from uninfected (*n* = 3) and infected RMs, with either SIVmac251 (*n* = 6) or SHIVSF162p3 (*n* = 6). After cell sorting of hepatic CD4^+^ T cells, viral DNA quantification and RNA sequencing were performed.Thus, we demonstrated that liver CD4^+^ T cells strongly expressed the SIV coreceptor, CCR5. We showed that viremia was negatively correlated with the percentage of hepatic effector memory CD4^+^ T cells. Consistent with viral sensing, inflammatory and interferon gene transcripts were increased. We also highlighted the presence of harmful CD4^+^ T cells expressing *GZMA* and members of *TGFB* that could contribute to fuel inflammation and fibrosis. Whereas RNA sequencing demonstrated activated CD4^+^ T cells displaying higher levels of mitoribosome and membrane lipid synthesis transcripts, few genes were related to glycolysis and oxidative phosphorylation, which are essential to sustain activated T cells. Furthermore, we observed lower levels of mitochondrial DNA and higher levels of genes associated with damaged organelles (reticulophagy and mitophagy). Altogether, our data revealed that activated hepatic CD4^+^ T cells are reprogrammed to lipid metabolism. Thus, strategies aiming to reprogram T cell metabolism with effector function could be of interest for controlling viral infection and preventing liver disorders.

**IMPORTANCE:**

Human immunodeficiency virus (HIV) infection may cause liver diseases, associated with inflammation and tissue injury, contributing to comorbidity in people living with HIV. Paradoxically, the contribution of hepatic CD4^+^ T cells remains largely underestimated. Herein, we used the model of simian immunodeficiency virus (SIV)-infected rhesus macaques to access liver tissue. Our work demonstrates that hepatic CD4^+^ T cells express CCR5, the main viral coreceptor, and are infected. Viral infection is associated with the presence of inflamed and activated hepatic CD4^+^ T cells expressing cytotoxic molecules. Furthermore, hepatic CD4^+^ T cells are reprogrammed toward lipid metabolism after SIV infection. Altogether, our findings shed new light on hepatic CD4^+^ T cell profile that could contribute to liver injury following viral infection.

## INTRODUCTION

CD4^+^ T cells expressing CCR5 represent the main population infected by human immunodeficiency virus (HIV) (1, 2). Furthermore, depletion of memory CD4^+^ T cells, which can be related to either immune activation or by bystander interaction with viral particles rendering the cells more prone to die, is associated with AIDS pathogenesis (3 – 5).

Since the 1990s, several reports indicated the presence of viral RNA in visceral tissues such as the liver (4, 6 – 8). Infection may cause liver diseases such as non-alcoholic fatty liver disease (9) and non-alcoholic steatohepatitis (10, 11), associated with inflammation and tissue damage (10, 12), particularly in individuals co-infected with hepatitis viruses (13) showing increased levels of alanine aminotransferase (ALT) and aspartate aminotransferase (AST) (14). Thus, an influx of CCR2-expressing cells into the liver of simian immunodeficiency virus (SIV)-infected rhesus macaques (RMs) has been reported (15), associated with an inflammatory gene signature (15). However, less attention has been paid regarding hepatic CD4^+^ T cells that may be related to human liver accessibility. *In vitro* studies have indicated that hepatocyte-bound virus allows viral transmission to CD4^+^ T cells (16, 17). Hepatic T cells have been observed to be infected *in situ* (15), while memory CD4^+^ T cells expressing CCR5 are reduced in the liver of SIV-infected RMs (18).

The inefficiency of the immune response in clearing microbes leading to chronic immune activation is associated with the exhaustion and the loss of effector T cells (19, 20). HIV/SIV infection is associated with exhausted CD8^+^ T cells characterized by the expression of programmed cell death 1 (PD-1) molecules (21, 22). Of interest, in the context of parasite infection, hepatic CD4^+^ T cells expressing PD-1 are associated with tissue damage (23). PD-1 expression is associated with T cell metabolic reprogramming by inhibiting glycolysis and promoting lipolysis and fatty oxidation (24). The importance of exhausted CD4+ T cells expressing PD-1 and their metabolism in the context of SIV infection is unknown so far.

We analyzed hepatic CD4^+^ T cells of SIV/SHIV-infected RMs. Thus, CD4^+^ T cells demonstrated high levels of viral DNA and displayed activated and interferon gene profiles. CD4^+^ T cells displayed a lipogenesis profile associated with membrane synthesis in the absence of those encoding for glycolysis or oxidative phosphorylation (25) that is consistent with the increased percentage of CD4^+^ T cells expressing PD-1 in SIV-infected RMs compared to non-infected RMs. Of importance, we found that CD4^+^ T cells express *GZMA,* which is considered to elicit inflammation (26), and *TGFBI,* which promotes fibrosis (27).

Altogether, we highlighted that SIV infection reprograms hepatic CD4^+^ T cell metabolism toward lipids in which the recruitment of CD4^+^ T cells could contribute to fuel inflammation in the liver of SIV-infected RMs.

## RESULTS

### Infection of hepatic CD4^+^ T cells is associated with the depletion of effector memory CD4^+^ T cells in SIV/SHIV-infected RMs

**Fig 1:**
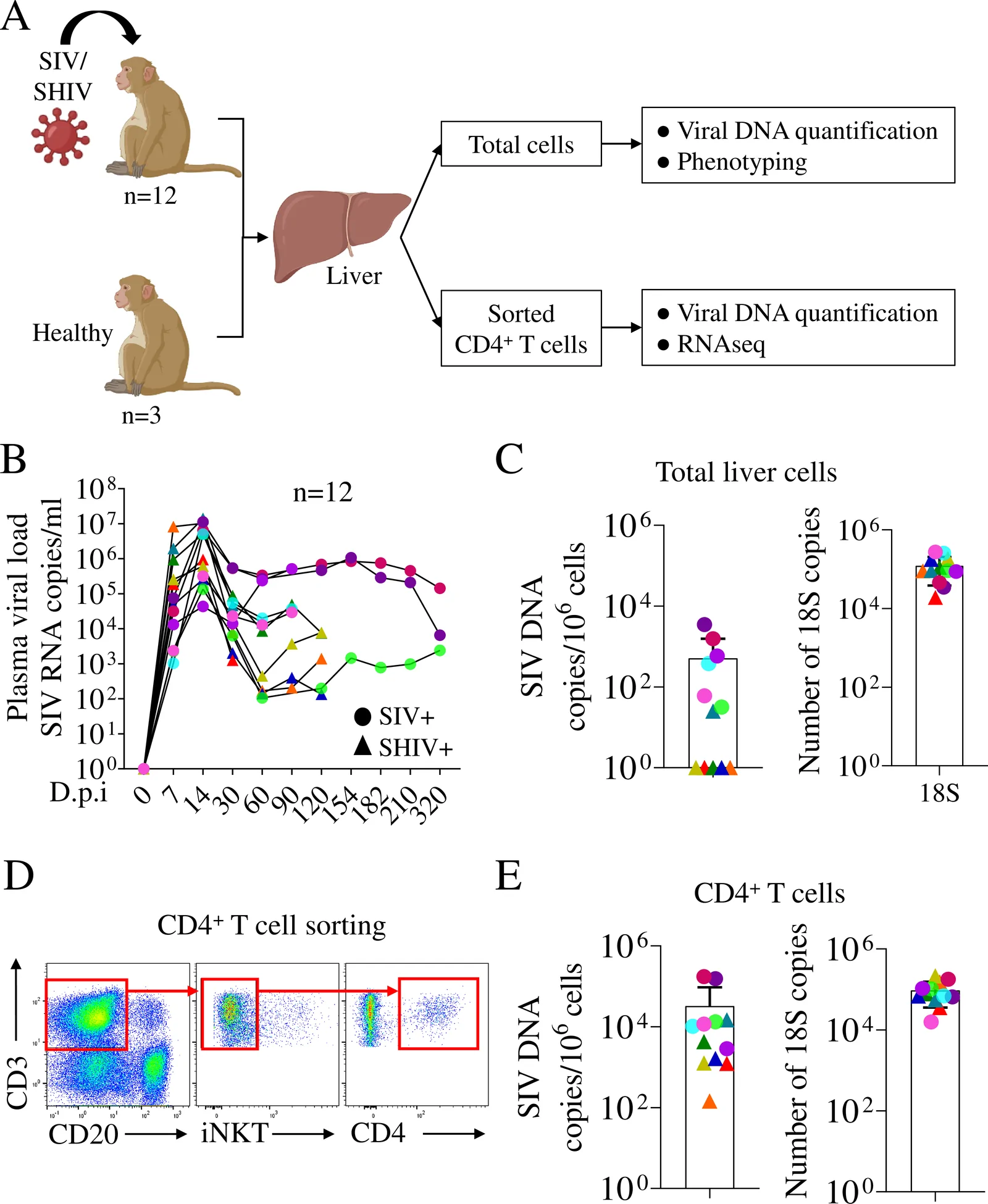
Schematic of the study protocol and viral dynamics in SIV/SHIV-infected RMs. (**A**) Rhesus macaques (RMs) are infected intrarectally with either SIVmac251 (*n* = 6) or SHIVSF162 (*n* = 6). Three healthy uninfected RMs are included as control group. Liver cells are recovered for viral DNA quantification, phenotyping, and cell sorting. RNA sequencing is performed from sorted hepatic CD4^+^ T cells. Figure created with BioRender.com. (**B**) Viremia is shown for each RM (expressed as copies per milliliter). (**C**) Left, frequencies of SIV DNA quantified in total liver cells (expressed as copies per 10^6^ cells). Right, number of 18S copies quantified. (**D**) Gating strategy of sorted CD4^+^ T cells. (**E**) Left, frequencies of cell-associated viral DNA. Right, number of 18S copies quantified. Each colored symbol represents one individual.

Twelve RMs of Chinese origin were infected with SIVmac251 (*n* = 6) and SHIVSF162p3 (*n* = 6). Animals were sacrificed at different time points post-infection during the chronic phase (Fig. 1A; Table S1). The peak of plasma viral load reached 7–14 days post-infection, ranging from 10^4^ to 10^7^ copies per milliliter (Fig. 1B). On the day of euthanasia, viremia ranged from 10^2^ to 10^5^ copies per milliliter (Table S1). Assessing viral seeding in the liver, cell-associated viral DNA was only detected in 7/12 RMs, reaching 10^1^ to 10^3^ copies per 10^6^ cells (Fig. 1C, left panel). The difference of viral DNA detected in the liver was not related to a difference in the quantity of cells analyzed, as shown by the expression of 18S DNA in Fig. 1C, right panel. We then sorted hepatic CD4^+^ T cells from the CD20^−^CD3^+^ T cell population by flow cytometry (Fig. 1D). However, we excluded invariant natural killer T (iNKT) cells (TCR Vα24-Jα18^+^) from cell sorting, since this innate T cells are enriched in the liver (28). Thus, we found that hepatic CD4^+^ T cells harbor 10^2^ to 10^5^ copies per 10^6^ cells (Fig. 1E, left panel) while similar levels of 18S DNA were detected (Fig. 1E, right panel). Interestingly, RMs in which we did not detect viral DNA in total liver (Fig. 1C, left panel) are those displaying the lower levels of viral DNA in hepatic CD4^+^ T cells. Thus, a positive correlation was observed (Fig. S1A). Enriching CD4^+^ T cells increased the sensitivity of viral detection by 10 to 100 times (Fig. S1B). Therefore, our results indicate viral seeding in the liver associated with CD4 T cell infection.

**Fig 2:**
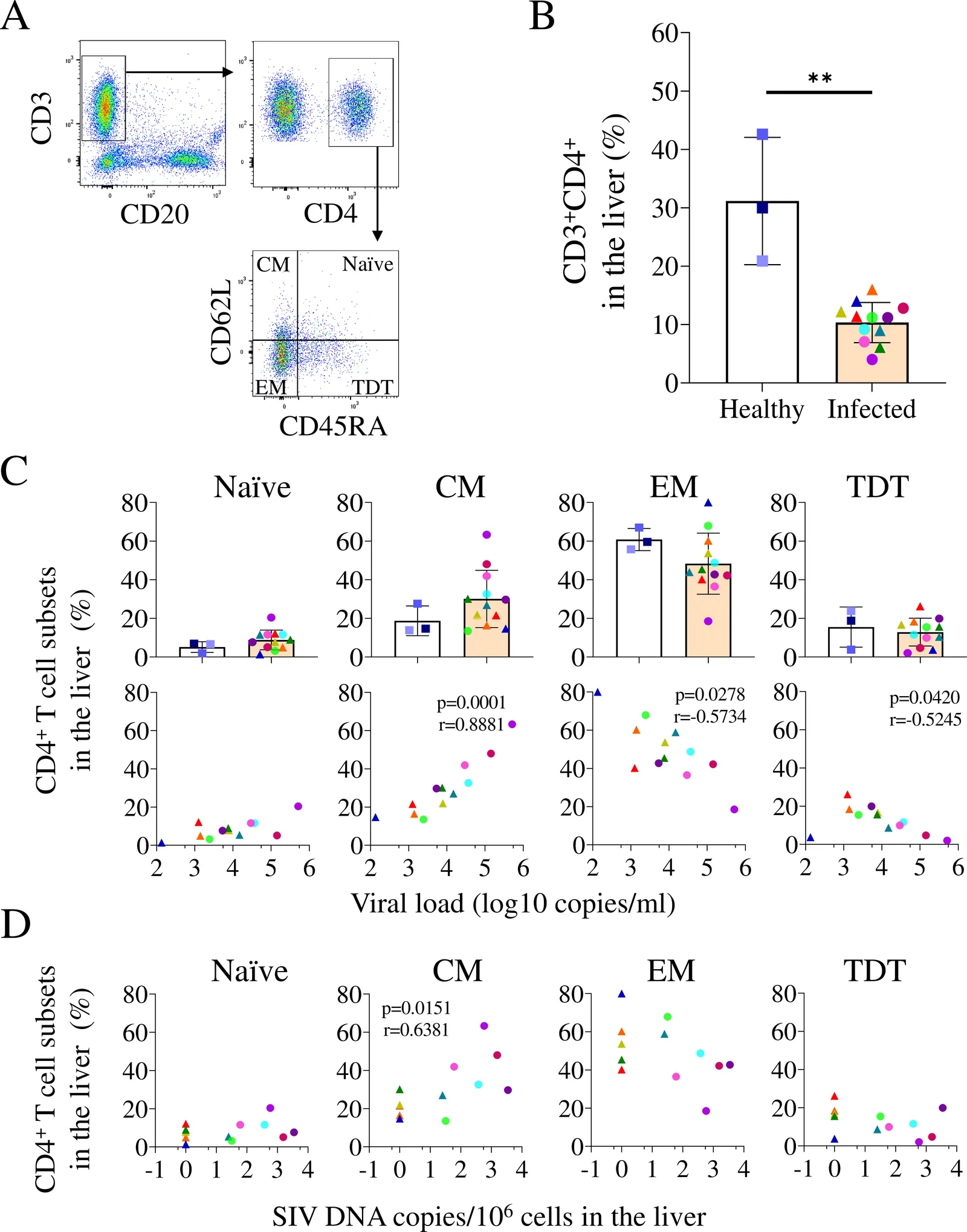
Hepatic CD4^+^ T cell dynamics. (**A**) Gating strategy depicting CD4^+^ T cell subsets based on the expression of CD62L and CD45RA. (**B**) Histograms show the percentages of hepatic CD3^+^CD4^+^ T cells of healthy uninfected RMs (*n* = 3) and SIV/SHIV-infected RMs (*n* = 12). (**C**) Top panel, histograms show the percentages of naïve (CD62L^+^CD45RA^+^), central memory (CM, CD62L^+^CD45RA^−^), effector memory (EM, CD62L^−^CD45RA^−^), and terminal differentiated (TDT, CD62L^−^CD45RA^+^) hepatic CD4^+^ T cells from healthy uninfected RMs (white) and SIV/SHIV-infected (orange) RMs. Bottom panel, correlations between the percentages of hepatic CD4^+^ T cell subsets and viral load. (**D**) Correlations between the percentages of hepatic CD4^+^ T cell subsets and frequencies of SIV DNA quantified in total liver cells. Spearman analysis was used to assess correlations. The R and *P*-values are indicated in the figures.

We then analyzed the dynamics of hepatic CD4^+^ T cells in comparison to those in peripheral blood. The gating strategy is shown in Fig. 2A. As expected, the percentages of blood CD4^+^ T cells were significantly decreased in SIV/SHIV-infected RMs (Fig. S2A). Interestingly, although the percentages of hepatic CD4^+^ T cells in non-infected RMs are lower to those observed in the blood (Fig. 2B; Fig. S2A), CD4^+^ T cells are also depleted in the liver of infected animals (non-infected, 31.17 ± 10.9; infected, 10.8% ± 3.6%; *P* = 0.0036) (Fig. 2B).

Based on the expression of CD62L and CD45RA molecules, discriminating naïve (CD62L^+^CD45RA^+^), central memory (CM, CD62L^+^CD45RA^−^), effector memory (EM, CD62L^−^CD45RA^−^), and terminal differentiated (TDT, CD62L^−^CD45RA^+^) cells, we assessed the dynamics of CD4^+^ T cell subsets (Fig. 2A). We found that CD4^+^ T cells in the liver are mostly effector memory T cells (Fig. 2C, top panel) compared to the blood (Fig. S2B), in which the pool of naïve CD4^+^ T cells is two- to threefold higher. This difference in the phenotype of hepatic versus blood CD4^+^ T cells suggests a minor contamination of blood cells in the analysis of liver cells. Whereas no significant difference was observed in the phenotype of hepatic CD4+ T cells between healthy and SIV-infected RMs (Fig. 2C, top panel), the percentages of EM and TDT are negatively correlated with plasma viremia (EM, *P* = 0.0278; TDT, *P* = 0.0420). Conversely, the percentages of CM are positively correlated with viral loads (*P* = 0.0001) (Fig. 2C, bottom panel). In the blood, while the percentages of EM are negatively correlated with plasma viremia (EM, *P* = 0.0264) (Fig. S2B), the percentages of naïve are positively correlated with viremia (naïve, *P* = 0.0150) demonstrating the distinct dynamics of CD4^+^ T cells in the liver, compared to the blood (Fig. S2B). Furthermore, we showed that the percentages of CM are positively correlated with the levels of SIV DNA in the liver (Fig. 2D). Conversely, the percentages of EM tend to be negatively correlated with viral DNA, although not statistically significant.

Thus, our results demonstrated that 0.1%–10% of hepatic CD4^+^ T cells are infected, associated with the depletion of effector memory CD4^+^ T cells.

### Transcriptomic signature of hepatic CD4^+^ T cells from SIV-infected RMs

**Fig 3:**
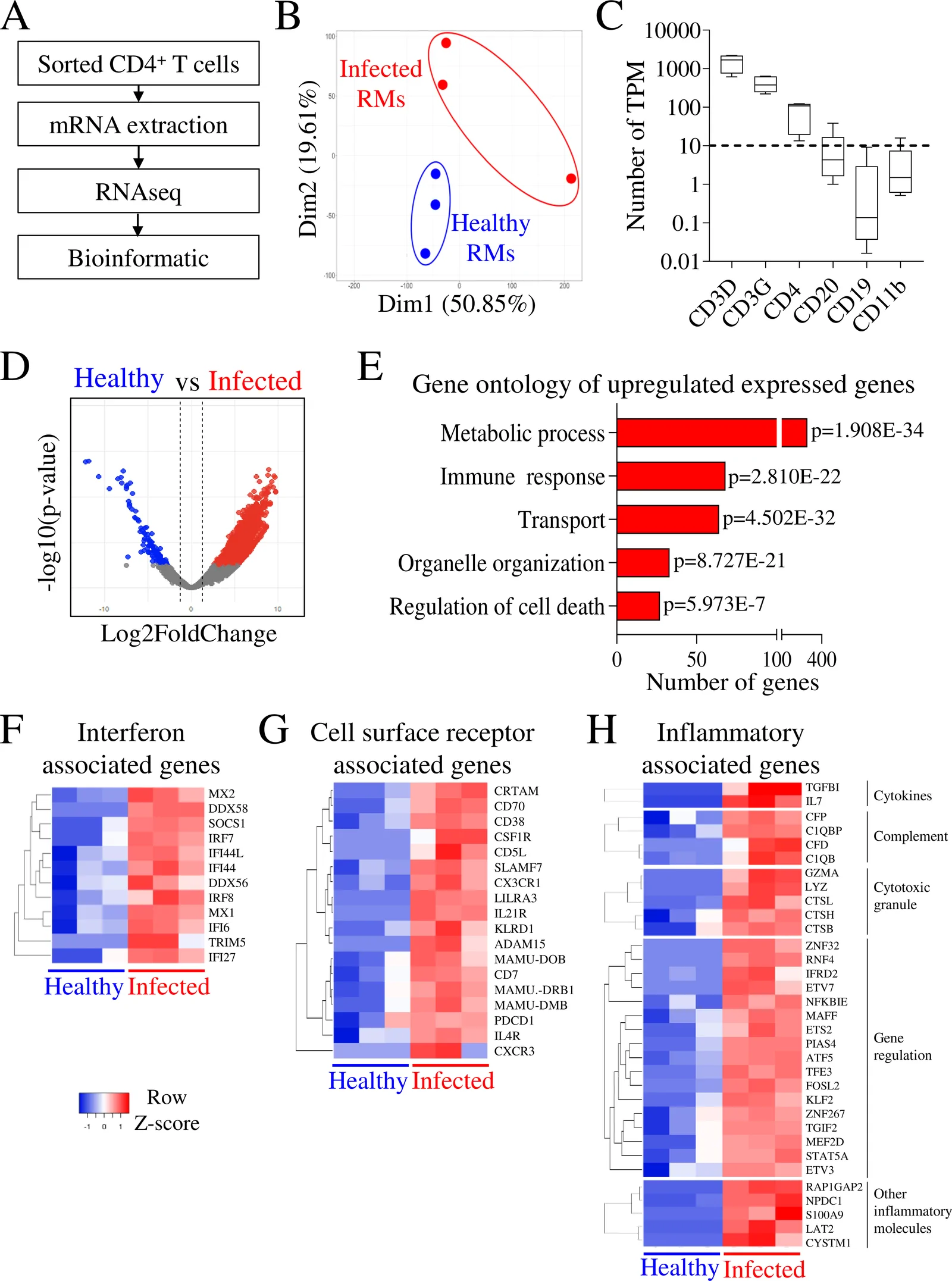
Transcriptomic signature of hepatic CD4^+^ T cells of SIV-infected RMs. (**A**) Experiment design for transcriptomic analyses of hepatic CD4^+^ T cells. (**B**) Principal component analysis showing the divergence of transcriptomic profiles of CD4^+^ T cells from healthy uninfected (*n* = 3, blue symbols) and SIV-infected RMs (*n* = 3, red symbols). Each cluster corresponds to the set of gene expressed. (**C**) Expression of genes specifically related to T (*CD3D*, *CD3G*, and *CD4*), B [*MS4A1* (CD20) and *CD19*], and myeloid (*ITGAM*, or CD11b) cells in sorted CD4^+^ T cells. Results are expressed as TPM count. Dashed line represents limit of transcript levels considered to be significant for differentially expressed gene analysis. (**D**) Volcano plot shows the distribution of differentially expressed genes in hepatic CD4^+^ T cells of uninfected and SIV-infected RMs. Red dots represent up-regulated genes and blue dots represent down-regulated genes. (**E**) Functional enrichment analysis showing gene ontology of up-regulated genes. Gene Ontology Biological Processes (GO_BP) are shown on the y-axis, and the number of genes involved in each GO_BP is shown on the x-axis. The *P*-values are also shown. (**F–H**) Heat maps represent the genes differentially expressed and associated with (**F**) interferon, (**G**) cell surface receptor, and (**H**) inflammation signatures from sorted hepatic CD4^+^ T cells from of healthy uninfected and SIV-infected RMs. Row z-score shows the differential expression of a single gene across the samples. Red bars indicate an increased abundance of the corresponding genes, and blue bars indicate a decreased abundance.

Gene profiles of hepatic CD4^+^ T cells have been poorly explored until now. Thus, a whole-transcriptomic analysis of sorted CD4^+^ T cells excluding iNKT cells from three SIV-infected RMs and three uninfected RMs was performed (Fig. 3A). Using a principal component analysis (PCA), transcriptomic profiles of CD4^+^ T cells were compared (Fig. 3B). Thus, Dim1 and Dim2 axes discriminated CD4^+^ T cells from healthy RMs to SIV-infected RMs, revealing the impact of viral infection on gene expression. CD4^+^ T cell-associated transcripts including *CD3D*, *CD3G*, and *CD4* are shown in comparison to either B cell-associated transcripts (*CD20* and *CD19*) or myeloid-associated transcript (*CD11b*) (Fig. 3B). Thus, consistent with the purity of cell sorting (higher than 96%), *CD3D*, *CD3G*, and *CD4* were highly enriched compared to *CD20*, *CD19,* and *CD11b* genes [that display lower than 10 transcripts per million (TPM) (Fig. 3C]. Thus, we selected transcripts having more than 10 TPM and having a log2FoldChange greater than 2.5 between uninfected and infected RMs. Volcano plot revealed up- and down-regulated genes (Fig. 3D). We found that 551 transcripts are up-regulated in CD4^+^ T cells while few transcripts are significantly down-regulated (Fig. 3D). Among these up-regulated transcripts, we found that they are clustered to metabolic process (Gene Ontology [GO]: 0008152, *P* = 1.908E−34), transport (GO: 0006810, *P* = 3.978E−32), immune response (GO: 0006955, *P* = 2.810E−22), organelle organization (GO: 0006996, *P* = 8.727E−21), and regulation of cell death pathway (GO: 0010942, *P* = 5.973E−7) (Fig. 3E). The genes are listed in Table S4.

Thus, specific transcriptomic signatures of hepatic CD4^+^ T cells characterize SIV-infected RMs.

### Transcriptomic data revealed activated and cytotoxic CD4^+^ T cells in the liver of SIV-infected RMs

We then assessed which genes are associated with the immune response pathway. Hepatic CD4^+^ T cells from SIVmac251-infected RMs demonstrated significantly higher levels of interferon-stimulated genes (ISG) including *IFI6/27/44/44L, IRF7/8, MX1/2, DDX56/58, TRIM5,* and the inducible negative regulator suppressor of cytokine signaling 1 (*SOCS1*) (Fig. 3F). This is consistent with viral sensing by infected CD4^+^ T cells. Furthermore, our results revealed a significant up-regulation of genes related to cell activation, such as major histocompatibility complex class II transcripts (*MAMU-DOB/DMB/DRB1*) and programmed death-1 molecule (*PDCD1*, PD-1) (Fig. 3G).

It is interesting to note that among the other genes, *CD38* is increased. CD4^+^ T cells expressing this ectoenzyme (CD38) have been reported to display immune regulatory activities such as cytotoxic (29) and suppressive functions (30). Thus, we found higher levels of *TGFBI* and its transcription factors *TGIF2* (TGFB-induced factor homeobox 2) (31, 32) (Fig. 3H). Whereas we did not find an increase of *GZMB* and *PRF1* transcripts, we found higher levels of *GZMA,* and three endopeptidases, cathepsins B, H, and L (*CTSB/H/L*), higher levels of *SLAMF7* (or CRACC, CD2-like receptor activating cytotoxic cells) (33 – 35), *CRTAM* (cytotoxic and regulatory T cell molecule) (36), *KLDR1* (killer cell lectin-like receptor D1) (37), and *ADGRG1* (GPR56) (38) suggesting the notion of cytotoxic CD4^+^ T cells (Fig. 3G through H). Our results also revealed higher levels of *IL7* transcripts and two transcription factors essential for Th1 polarization (39), *KLF2* and *STAT5,* this latter being related to the interleukin (IL)-7 signaling pathway (40) and to CD4^+^CD38^+^ T cells (29, 41).

Our results also demonstrated inflamed CD4^+^ T cells highlighted by the expression of several inflammatory genes including *CX3CR1* and *CXCR3* transcripts. Indeed, in inflamed tissues, activated T cells expressing CXCR3 are recruited via CXCL10 (interferon protein-10), a chemokine increased in the liver of SIV-infected RMs (Clain et al., submitted for publication). *CX3CR1* expression reflects highly differentiated CD4^+^ T cell subset reported to be present in inflamed tissues (42 – 44) (Fig. 3G). Other genes also indicated inflamed T cells such as *CD70* (Tnfsf7) in which CD70 promoter is demethylated leading to gene expression in the context of several autoimmune and inflammatory diseases (45, 46). Similarly, *SLAMF7* expression was reported to be associated with inflammatory diseases and tissue lesions driving an IgG4 humoral response (33, 47, 48), which is generally associated with Th2 cells (49, 50). Herein, the levels of *IL4R* and *IL21R* transcripts are increased in hepatic CD4^+^ T cells from SIV-infected RMs (Fig. 3G). Surprisingly, we also detected genes related to complement systems (*CFD, CFP, C1QB, C1QBP*) that have been previously implicated in tissue lesions (51) (Fig. 3H). Table S5 indicates the top 10 of differentially expressed genes for each pathway. The log2FoldChange and associated *P*-values are also indicated.

Altogether, our data demonstrated that hepatic CD4^+^ T cells exhibit an ISG signature reflecting viral detection in which a strong signature is related to inflamed activated cytotoxic CD4^+^ T cells.

### Increase of PD-1 expressing CD4^+^ T cells in the liver of SIV/SHIV-infected RMs

**Fig 4:**
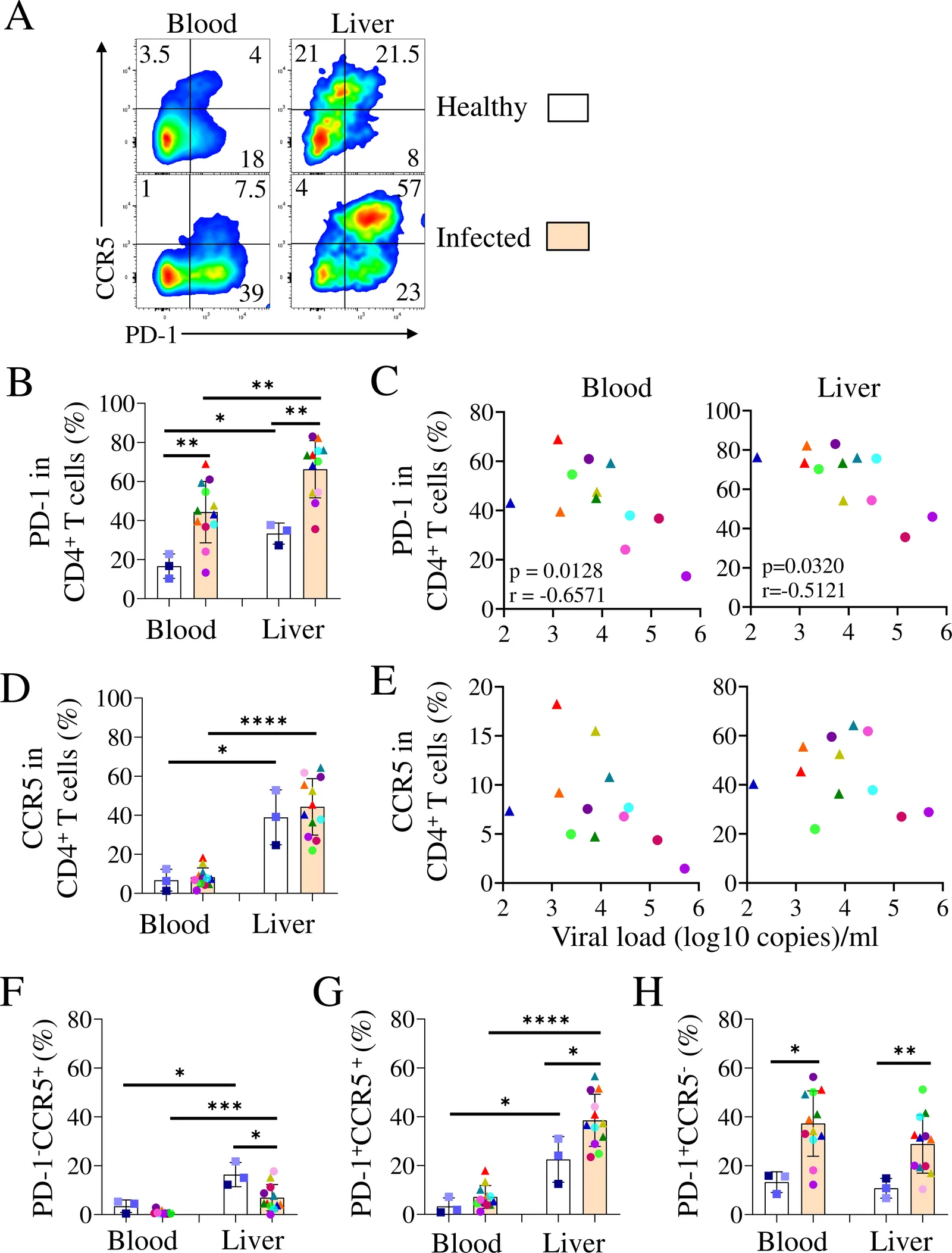
Expression of CCR5 and PD-1 of CD4^+^ T cells from the blood and liver of healthy uninfected and SIV/SHIV-infected RMs. (**A**) Representative dot plots depicting the expression of CCR5 and PD-1 on CD4^+^ T cells from the blood and liver of healthy uninfected (top) and SIV/SHIV-infected (bellow) RMs. (**B**) Histograms show the percentage of PD-1 on CD4^+^ T cells from the blood and liver of healthy uninfected (white) and SIV/SHIV-infected (orange) RMs. (**C**) Correlations between viral load and the percentage of PD-1 on CD4^+^ T cells from the blood and liver of SIV/SHIV-infected RMs. (**D**) Histograms show the percentage of CCR5 on CD4^+^ T cells from the blood and liver of healthy uninfected and SIV/SHIV-infected RMs. (**E**) Correlations between viral load and the percentage of CCR5 on CD4^+^ T cells from the blood and liver of infected RMs. (**F–H**) Histograms show the percentage of (**F**) PD-1^−^CCR5^+^, (**G**) PD-1^+^CCR5^+^, and (**H**) PD-1^+^CCR5^−^ cells on CD4^+^ T cells from the blood and liver of healthy uninfected (white) and SIV/SHIV-infected (orange) RMs. Statistical analysis was performed using a Mann-Whitney test. *, *P* < 0.05; **, *P* < 0.01; ***, *P* < 0.001; ****, *P* < 0.0001. Spearman analysis was used for correlations. The *r-* and *P*-values are indicated in the figures.

Because hepatic CD4^+^ T cells are infected, we evaluated the expression of CCR5, the main coreceptor for SIV (2), and of the checkpoint inhibitor PD-1 molecule (21, 22) as revealed by transcriptomic analysis. However, PD-1 expression may also indicate an early T cell immune activation (52) in which some cytokines such as TGF-β increase its cell surface expression (53). Flow cytometry was used to assess the expression of PD-1 and CCR5 (Fig. 4A) from hepatic and blood CD4^+^ T cells.

First, we observed higher levels of PD-1 expression on CD4^+^ T cells from infected RMs both in blood (healthy, 16.6% ± 6.3% versus infected, 44.3% ± 15.7%; *P* = 0.0088) and liver (healthy, 33.3% ± 5.4% versus infected, 66.3% ± 14.7%; *P* = 0.0044) compared to non-infected RMs (Fig. 4B). Of interest, our data highlighted that the levels of CD4^+^PD-1^+^ cells are negatively correlated with viremia, both in the liver and blood (liver, *P* = 0.0128; blood, *P* = 0.0320) (Fig. 4C).

Second, our results indicated that the percentage of CD4^+^ T cell expressing CCR5 in the liver is extremely elevated compared to that observed in blood CD4^+^ T cells (Fig. 4D through E) (healthy: blood, 6.7% ± 5.6% versus liver, 38.9% ± 14.0%; *P* = 0.0112). This difference in the phenotype of hepatic versus blood CD4^+^ T cells further supports a minor contamination of blood CD4^+^ T cells in the analyses of liver cells. However, we did not observe a difference in the percentage of CCR5^+^CD4^+^ T cells between infected versus non-infected RMs (Fig. 4D) as well as with the extent of viremia (Fig. 4E). This may reflect the difference in the dynamics of CCR5 in Chinese RMs in which the depletion of CCR5^+^CD4^+^ T cells is less, and the expression is even increased, due to immune activation and compared to what has been reported in RMs of Indian origin (2). Thus, we analyzed the co-expression of PD-1 and CCR5.

Whereas PD-1^−^CCR5^+^ CD4^+^ T cells in the liver are significantly decreased in SIV/SHIV-infected RMs (6.9% ± 5.4%) compared to uninfected (16.4% ± 4.9%; *P* = 0.0242) (Fig. 4F), the PD-1^+^CCR5^+^ population is, on the contrary, significantly increased, as shown in Fig. 4G (healthy, 22.5% ± 9.3% versus infected, 38.54% ± 10.7%; *P* = 0.0242). In the blood, we observed the same tendency (PD-1^−^CCR5^+^, healthy, 3.4% ± 2.6% versus infected, 0.9% ± 0.8%; PD-1^+^CCR5^+^, healthy, 3.3% ± 3.4% versus infected, 7.1% ± 4.6%). The percentage of PD-1^+^CCR5^−^ CD4^+^ T cells is significantly increased both in the blood and liver (Fig. 4H).

By analyzing the expression of CCR5 in CD4^+^ T cell subsets, we showed that levels of CM expressing CCR5 are positively correlated with plasma viremia, whereas the levels of EM expressing CCR5 are negatively correlated (Fig. S3A). Similarly, we observed a positive correlation between the percentages of CM expressing PD-1 and viral loads, while we found a negative correlation between the percentages of EM expressing PD-1 and viremia (Fig. S3B). In the blood, whereas no correlation was observed for CCR5 expressing cells (Fig. S3C), only EM levels expressing PD-1 are negatively correlated with viremia (Fig. S3D).

Altogether, our results demonstrated that hepatic CD4^+^ T cells express higher levels of PD-1 in SIV-infected RMs in which the infection has a minor impact on the depletion of CD4^+^ T cells expressing CCR5, reflecting the balance between activation and depletion, as previously reported in RMs of Chinese origin (2).

### Enriched autophagic gene signature in hepatic CD4^+^ T cells from SIV-infected RMs

**Fig 5:**
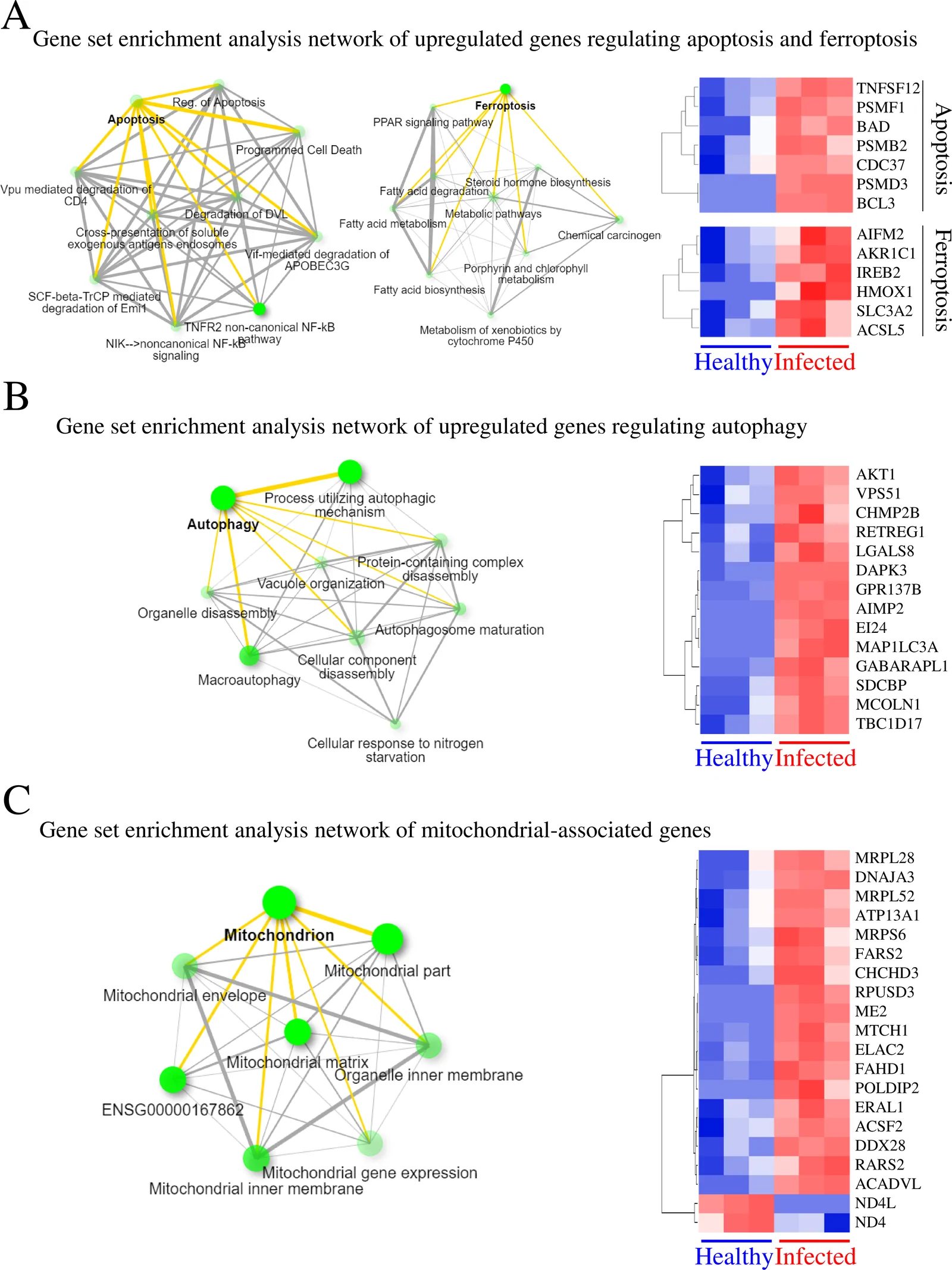
Apoptosis, ferroptosis, autophagy, and mitochondrial transcripts in hepatic CD4^+^ T cells of SIV-infected rhesus macaques. (**A**) Gene-set clustering analysis (left) and heatmaps (right) show an enrichment of genes related to the regulation of apoptosis and ferroptosis pathways. (**B**) Gene-set clustering analysis (left) and heatmap (right) show an enrichment of genes regulating autophagy. (**C**) Gene-set clustering analysis (left) and heatmap (right) show an enrichment of genes related to mitochondria. Two pathways (green nodes) are connected if they share 20% or more genes. Larger dots indicate more significant *P*-values. Thicker edges represent more overlapped genes. For heatmaps, row z-score shows the differential expression of a single gene across the samples. Red bars indicate an increased abundance of the corresponding genes, and blue bars indicate a decreased abundance.

Having observed that most of the CD4^+^ T cells express PD-1, initially demonstrated to be associated with cytokine withdrawal (54) that can lead to apoptosis and autophagy, we then analyzed the profile of these genes that can be differentially expressed in hepatic CD4^+^ T cells, from SIV-infected RMs. Transcriptomic analyses indicated the up-regulation of several genes associated with apoptosis (Fig. 5A). Whereas the pro-apoptotic master regulators, Bak and Bax (55, 56), are not up-regulated, we found higher levels of *BAD* related to cell death mediated by cytokine withdrawal (57). The other genes identified are related to NF-κB activation. *BLC3* that targets p50 homodimers of NF-κB (58, 59) is a coactivator of NF-κB-dependent transcription. Cyclin-dependent kinase 37 (*CDC37*), a chaperone of Hsp90, which interacts with the inhibitor of NF-κB kinase (IKK) (60) and with most of the kinome. Our results also highlighted the up-regulation of several ferroptosis genes, including the heme oxygenase-1 (*HMOX1*) (61), the iron-responsive element-binding protein 2 (*IREB2*) involved in the modulation of iron transporters (62), and the solute carrier family 3 member 2 (*SLC3A2*), a subunit of system X_C_- (63). However, higher levels of ferroptosis-protective genes, namely the ferroptosis suppressor protein 1 (FSP1, previously known as *AIFM2*) (64, 65) and the aldo-keto reductase 1C (*AKR1C1*) are observed.

We also found that hepatic CD4^+^ T cells from infected animals expressed autophagic-associated genes (Fig. 5B) such as *DAPK3,* an autophagy regulator (66), *AKT1* that relies on the PI3K pathway, and the MDA-9/syntenin (Melanoma differentiation-associated gene-9/syntenin) that also regulates PI3K and autophagy (67). We also identified genes related to endosome (*VPS51* and *CHMP2b*), and lysosome dynamic and integrity including reticulophagy and mitophagy processes [*GPR137B, RETREG1 (or FAM134B), MAP1LC3A, TBC1D17, LGALS8,* and the autophagy-related Atg8 protein *GABARAPL1*] (68 – 70). Furthermore, we also identified genes related to protein that facilitates Ca^2+^ transfer such as mucolipin 1 (*MCOLN1/TRPML1*) and *EI24* (etoposide-induced protein 2.4) that is enriched at the endoplasmic reticulum (ER)-mitochondria interface (71). The log2FoldChange and *P*-value of the top 10 of differentially expressed genes are indicated in Table S5.

Therefore, our results indicated that autophagy is the main signature in hepatic CD4^+^ T cells, compared to apoptosis and ferroptosis, although they can be intertwined.

### Mitoribosome machinery signature characterizes hepatic CD4^+^ T cells from SIV-infected RMs

Because we identified critical genes related to mitophagy machinery that can be related to energetic stress, we analyzed the genes associated with mitochondria. We found that overexpressed genes (Fig. 5C) include mitochondrial ribosomal protein transcripts (*MRPL28, MRPL52, MRPS6*), metabolism-associated transcripts such as *FARS2* (phenylalanyl-tRNA) and *RARS2* (arginyl-tRNA) synthetases, *RPUSD3* (RNA pseudouridine synthase D3), *ERAL1* (Era-like 12S mitochondrial RNA chaperone 1), *ELAC2* (mitochondrial RNase Z), and *DDX28* (mitochondrial DEAD-box protein) that contribute to the formation of the mitoribosome. We also observed an up-regulation of mitochondrial metabolism-associated transcripts, such as *ACSF2* (acyl-CoA synthetase family member 2), *ACADVL* (very long-chain specific acyl-CoA dehydrogenase), *FAHD1* (fumarylacetoacetate hydrolase domain containing protein 1), *ME2* (the mitochondrial NADP^+^-dependent malic enzyme, an oxidative decarboxylase), *POLDIP2* (DNA polymerase delta-interacting protein 2), the chaperone transcript heat shock *DNAJA3,* and a component of MICOS (mitochondrial contact site and cristae organizing system), *CHCHD3*. However, we only observed one transcript related to the tricarboxylic acid cycle (*SDHC*), that is part of the mitochondrial succinate dehydrogenase complex involved in the oxidation of succinate to fumarate. We did not observe higher levels of transcripts related to the oxidative phosphorylation (OXPHOS) machinery or glycolysis that are generally associated with T cell activation and proliferation (72), but the down-regulation of the NADH dehydrogenase-associated transcripts ND4 and ND4L (Fig. 5C). These genes are encoded by mitochondrial DNA and represent two main components of the complex I of the OXPHOS complex located in the inner membrane of the mitochondria. Such alteration in gene expression has been associated with defective mitochondria and bioenergetic (72), in which mitophagy is essential for removing damaged mitochondria.

Altogether, our results demonstrated higher levels of transcripts related to mitochondrial RNA machinery, consistent with activated CD4^+^ T cells but altered mitochondria, due to the lower levels of ND4/ND4L that may contribute in turn to induce genes related to cellular quality control such as reticulophagy and mitophagy.

### Reprogramming into lipid metabolism of hepatic CD4^+^ T cells from SIV-infected RMs

**Fig 6:**
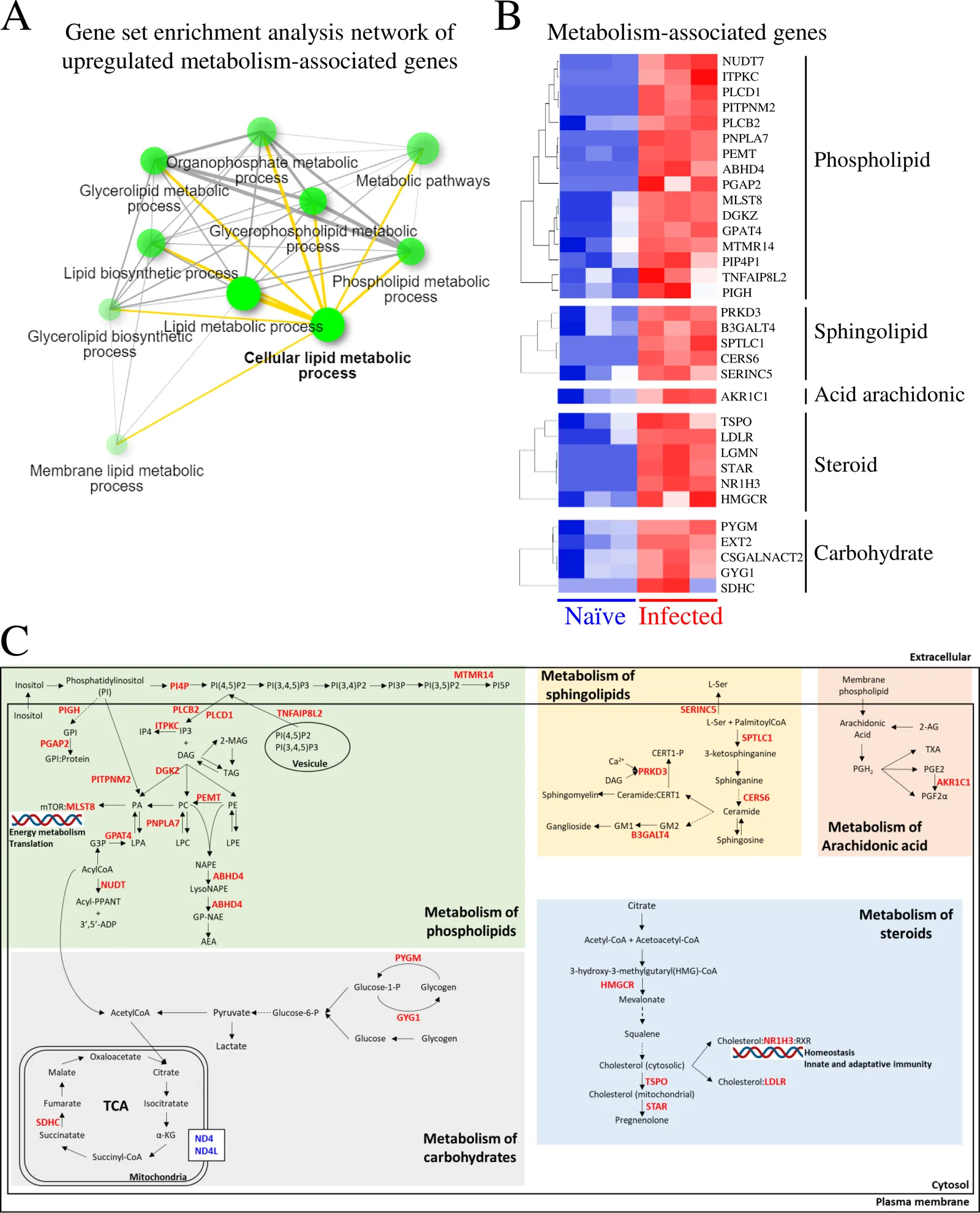
Metabolic transcriptomic profile of hepatic CD4^+^ T cells in SIV-infected RMs. (**A**) Gene-set clustering analysis shows an enrichment of genes related to the cellular lipid metabolic process. Two pathways (green nodes) are connected if they share 20% or more genes. Bigger dots indicate more significant *P*-values. Thicker edges represent more overlapped genes. (**B**) The heat maps represent the differential expression of genes associated to phospholipid, sphingolipid, acid arachidonic, steroid, and carbohydrate metabolism, in hepatic CD4^+^ T cells of healthy uninfected and SIV-infected RMs. Row z-score shows the differential expression of a single gene across the samples. Red bars indicate an increased abundance of the corresponding genes, and blue bars indicate a decreased abundance. (**C**) Schematic metabolic pathways showing up-regulated (red) and down-regulated (blue) transcripts in hepatic CD4^+^ T cells of SIV-infected RMs.

In addition to PD-1 that has been reported to alter cell metabolism by inhibiting glycolysis and promoting lipolysis/fatty acid oxidation (24), we also found higher levels of *CD5L* transcript, which regulates fatty acid composition and cholesterol biosynthesis in Th17 (73). Our results indicated an enrichment of transcripts associated with cellular lipid metabolic process (Fig. 6A). Most up-regulated transcripts are related to phospholipid, sphingolipid, acid arachidonic, and steroid metabolism (Fig. 6B). Interestingly, we also showed a series of transcripts involved in membrane synthesis such as ceramide, sphingosine, ganglioside, and serine (*SPTLC1, CERS6, PRKD3, B3GALT4, SERINC5*), phosphatidylinositol (*PI4P, PIGH, PGAP2, MTMR14, TNFAIP8L2, PLCD1, PLCB2, ITPKC*), phosphatidic acid (*DGKZ, PITPNM2, GPAT4, NUDT*), and phosphatidylcholine (*PEMT, PNPLA7*). We also found transcripts involved in citrate (*HMGCR*) and several genes related to cholesterol metabolism such as *TSPO, STAR, NR1H3, and LDLR*.

On the contrary, we observed few genes associated with carbohydrate metabolism in hepatic CD4^+^ T cells from SIV-infected RMs (Fig. 6B and C). Thus, several transcripts are related to glycogen (*PYGM* and *GYG1*) and glycosaminoglycan metabolism (*CSGALNACT2* and *EXT2*).

Taken together, our results strongly suggest that the metabolism of hepatic CD4^+^ T cells is reprogrammed toward a more pronounced lipid and membrane metabolism signature.

## DISCUSSION

Consistent with viral sensing, we have demonstrated that hepatic CD4^+^ T cells in SIV-infected RMs display interferon and inflammatory gene signatures. Our results highlighted that hepatic effector memory CD4^+^ T cells are depleted, while central memory cells are increased, in proportion to the extent of viremia, displaying higher levels of CCR5 and PD-1. Transcriptomic analyses revealed activated CD4^+^ T cells (human leukocyte antigen [HLA]-DR transcripts) associated with higher levels of mitoribosome and cholesterol synthesis transcripts, which have long been recognized to be necessary for cell growth and proliferation (74 – 76). We also found a strong enrichment of genes that dynamically regulate ER, mitochondria, and lysosomal machinery (reticulophagy and mitophagy). Contrasting with lipid signature, we did not find any increase in the transcripts encoding for glycolysis and oxidative phosphorylation machinery. Furthermore, we observed a decrease in transcripts encoded by mitochondrial DNA. Thus, a major reprogramming of CD4^+^ T cells toward a more pronounced lipophagy and lysosomal lipolysis is observed (77). This could be consistent with higher levels of PD-1 expression that promote lipolysis in T cells (24) and of *CD5L* transcripts, which regulate fatty acid composition and cholesterol biosynthesis (73). Finally, our results indicated a strong enrichment of genes encoding for cytotoxic transcripts that may participate in tissue injury and fibrosis. Altogether, our results highlighted the presence of activated metabolic reprogrammed CD4^+^ T cells in the liver of SIV-infected RMs.

An increasing body of evidence suggests that a coordinated rewiring of cellular metabolism is required to fulfill the bioenergetic and biosynthetic demands of T cells, following activation (78). Whereas naïve T cells predominantly rely on fatty acid β-oxidation and oxidative phosphorylation for their energy supply in the quiescent state, T cell activation induced metabolic reprogramming (78). However, in the absence of extrinsic signals, cell viability is compromised by insufficient nutrient utilization (79). Cholesterol has long been recognized to be an important component of cell membranes and an absolute requirement in cell cycle progression, cell growth, and proliferation (74 – 76). Therefore, the requirement for adequate cholesterol is obvious in activated CD4^+^ T cells (80, 81). While it has been shown that Th1 and Th17 cells are highly glycolytic T cells, Treg cells preferentially use lipid oxidation (82 – 84). Thus, distinct metabolism programming characterizes T cell profiles. Herein, we found that most of the genes associated with cellular metabolism are related to lipid and membrane synthesis, suggesting a major reprogramming of hepatic CD4^+^ T cells toward a more pronounced lipophagy and lysosomal lipolysis (77) instead of a glycolytic and/or oxidative phosphorylation metabolic pathway, following SIV infection. This could be consistent with a previous report indicating that higher levels of PD-1 expression promote lipolysis in T cells (24). Interestingly, we found an increase of *ACADVL* that catalyzes the initial step of mitochondrial β-oxidation and target esters of long-chain fatty acids as an attempt to re-establish lipid homeostasis and limit lipotoxicity (85). Enhancing *ACADVL* expression may improve the ability of hepatic liver CD4^+^ T cells to respond to metabolic stress and, consequently, improve their ability to persist and survive, even in a metabolically unfavorable environment (86). This can be consistent with the observation that several non-canonical autophagic genes (reticulophagy and mitophagy) are increased and may provide protection by eliminating damaged organelles and a bioenergetic supply for activated T cells (72). Thus, our findings unveil the metabolic signature of hepatic CD4^+^ T cells and provide evidence of how these cells are reprogrammed during an SIV infection.

Cytotoxic CD4^+^ T cells have been reported to have critical effector functions in the context of several pathologies, such as in the elimination of melanoma tumor cells (87, 88), in the protection against hepatocellular carcinoma (89), and in the control of several viral infections (90 – 92). Our analyses highlighted the expression of cytotoxic biomarkers such as *CD38* (29), *SLAMF7* (33 – 35), *CRTAM* (36), *KLDR1* (37), *ADGRG1* (38), and of endopeptidases (*CTSB/H/L*) suggesting that hepatic CD4^+^ T cells acquire a cytotoxic profile in response to SIV infection. However, *PRF1* and *GZMB* (93) are not increased, but *GZMA* is. Whereas GzmB is clearly considered to be a cytotoxic molecule, the pro-apoptotic effect of GzmA was mostly observed at a non-physiological level. Thus, the role of GzmA is considered to elicit inflammation (26) and promote a non-apoptotic inflammasome pathway, leading to bioactive IL-1β (94, 95). Therefore, this population of CD4^+^ T cells could contribute to fueling inflammation and liver injury instead of controlling viremia. Whether this population of CD4^+^ T cells can be reprogrammed to express *GZMB* and *PRF1* instead to *GZMA* merits to be further explored.

We also noticed that hepatic CD4^+^ T cells also overexpressed *CX3CR1* and *CXCR3* transcripts, suggesting the recruitment of this population in inflamed tissues (42, 43) and consistent with higher levels of *CXCL10* transcripts (the ligand of CXCR3) observed in the liver of SIV-infected RMs (96). Interestingly, CXCL10 was reported to be associated with liver fibrosis in individuals co-infected with HIV and Epstein–Barr virus (EBV) (97). In HIV-infected individuals co-infected with hepatitis viruses, the levels of AST and ALT are elevated. These biomakers have been associated with fibrosis, although imperfect measurements of hepatic fibrosis (14). Here, hepatic CD4^+^ T cells from SIV-infected RMs express *TGFBI* and *TGIF2* transcripts, which are associated with fibrosis (27). However, we did not assess the plasmatic levels of AST and ALT in SIV-infected RMs, which should be of interest.

One limitation of our study is that transcriptomic analyses are only performed on SIVmac251-infected RMs. Therefore, the gene profiles of hepatic CD4^+^ T cells from SHIV-infected RMs should be further addressed to see if they are similar, since these animals demonstrated lower viremia. Because our study does not include blood CD4^+^ T cell analysis, we cannot exclude that hepatic inflamed CD4^+^ T cells are recruited from blood or acquired novel effector functions, due to local hepatic environments participating in tissue damage. Indeed, in a recent study (Clain et al., in press), we found that transcripts related to inflammation, metabolism alteration, and tissue damage are increased in the liver of viremic SIV-infected RMs.

Whereas transcriptomic analyses have provided strong signatures of hepatic CD4^+^ T cells from SIV-infected RMs, gene expressions are not obligatorily associated with protein expressions. Thus, a limitation of our study is the absence of functional metabolism assessment. Nevertheless, the observation of genes related to mitophagy and reticulophagy associated with lower mtDNA indicated that mitochondria can be altered (71). Indeed, we and others have reported that the alteration of mitochondria shaping is associated with a lower respiratory capacity and mitochondrial membrane potential loss, leading in turn to mtDNA instability (98 – 100). Mitochondria alteration in CD4^+^ T cells from HIV-infected individuals (101 – 104) and in SIV-infected RMs (105) has been reported earlier and associated with a higher propensity of CD4^+^ T cells to die and to develop AIDS (3 – 5).

In this study, we used RM models to access hepatic tissues to highlight virological events associated with CD4^+^ T cells during SIV/SHIV infection. Thus, we demonstrated the reprogramming of activated CD4^+^ T cells toward lipid and cholesterol metabolisms associated with PD-1 expression. Furthermore, our data also raised the question of the harmful role of activated CD4^+^ T cells that could participate to liver inflammation in SIV-infected RMs. Thus, a strategy aiming to reprogram hepatic CD4^+^ T cells and/or limit their recruitment could be beneficial.

## MATERIALS AND METHODS

### Animals, viral inoculation, and sample collection

Twelve rhesus macaques seronegative for SIV, STLV-1 (simian T leukemia virus type 1), SRV-1 (simian type D retroviruses), and (type D retrovirus), and herpes B viruses were infected intrarectally with SIVmac251 (*n* = 6) and SHIVSF162p3 (*n* = 6) at the dose of 20AID_50_. RMs were sacrificed at different time points post-infection, as shown in Table S1. In comparison, three uninfected animals were also sacrificed. Immediately after euthanasia, liver cells were isolated by mechanical process for phenotypic analysis and cell sorting by flow cytometry. Tissues were not digested with collagenase or other proteases to limit the negative effects on the expression of cell surface markers. Aliquots of total liver cells were supplied for cell-associated viral DNA quantification. Total blood cells in EDTA were used for phenotypic analysis.

### Immunophenotyping and cell sorting

Fresh cells from the liver and peripheral blood collected from EDTA-coated tubes were stained with monoclonal antibodies in the dark for 30 min at 4°C. The fluorochrome-conjugated antibodies used are provided in Table S2. Cells were washed twice in phosphate-buffered saline (PBS) by centrifugation at 1,500 rpm for 7 min. Cells were washed and resuspended in cold paraformaldehyde phosphate buffer (PFA) 1%, after lysing erythrocytes (Lysing buffer Pharm Lyse, BD Biosciences) for 15 min. A total of 60,000 events corresponding to mononuclear cells were recorded on a BD FACSCelesta (BD Biosciences). Analyses were performed using Flowjo software (Tree Star, Inc.). Cells (10^8^ cells) were sorted using a BD Influx cell sorter (Becton Dickinson) using specific antibodies: Anti-CD3, CD4, CD20, and TCR Vα24-Jα18 mAbs (Table S2). A cell sorting strategy privileging purity (“purity mode”) versus enrichment is used, leading to a high level of purity which is always higher than 96%.

### Viral RNA quantification

As previously described (106, 107), viral loads in the sera of SIV- and SHIV-infected RMs were quantified by RT-qPCR using a PureLink Viral RNA/DNA Kit (Invitrogen). The PCR mixture comprises 4× TaqMan Fast Virus 1-Step Master Mix (Applied Biosystems), 750 nM of primers and 200 nM of probe. Primers and probe sequences are listed in Table S3. Serial 10-fold dilutions of a plasmid encoding for gag gene of SIVmac251 were performed to generate a standard curve. Amplifications were performed with a QuantStudio 6 Flex Real-Time PCR System (Applied Biosystems), using the following parameters: 50°C/5 min, 95°C/20 s, and 40 cycles (95°C/15 s, 60°C/1 min). Samples were run in duplicate, and results expressed as SIV RNA copies per milliliter.

### Cell-associated viral DNA quantification

As previously described (106, 107), quantification of cell-associated viral DNA was performed from 10^5^ sorted cells. For DNA extraction, we used the Genomics DNA Tissue kit (Macherey Nagel). DNA was eluted with 50 µL of elution buffer, and 10 µl of DNA eluate was amplified by nested PCR with SIVmac251- and SHIVSF162P3-specific primers surrounding the nef coding region. Primers are listed in Table S3. Using 50 nM of primers, 10× PCR buffer, 2 mM MgCl2, 1.25 U of AmpliTaq (Applied Biosystems), and 0.8 mM DNTP (Invitrogen), a first round of PCR was performed in a Biometra thermocycler, using the following parameters: 95°C/1 min 45 s, 20 cycles (95°C/30 s, 60°C/30 s, and 72°C/1 min 10 s), and 72°C/6 min. Five microliters of PCR product were re-amplified in a QuantStudio 6 Flex Real-Time PCR Systems (Applied Biosystems) using 250 nM of SIV DNA primers and probe (Table S3) and 2× PrimeTime Gene Expression Master Mix (IDT). The following parameters were used for the amplifications: 95°C/3 min and 45 cycles (95°C/15 s, 60°C/1 min). Samples were run in quadruplicate, and results are expressed as number of SIV DNA copies per 10^6^ cells. Serial dilutions of a plasmid were performed to generate a standard curve.

### RNA sequencing

RNA sequencing was performed using the Qiagen RNeasy Micro Kit. Total RNA was extracted from samples preserved in TRIzol with DNaseI treatment following the manufacturer’s protocol. Quantity and quality of total RNA were assessed by NanoDrop ND-1000 spectrophotometer (NanoDrop Technologies, Wilmington, DE, USA) and Bioanalyzer 2100 (Agilent Technologies, Santa Clara, CA, USA). Using the NEBNext Ultra II directional RNA Library Prep Kit for Illumina (New England Biolabs Inc., Ipswich, MA, USA), the mRNA sequencing libraries were prepared according to the manufacturer’s instructions. Using the NEBNext poly(A) (New England Biolabs Inc., Ipswich, MA, USA), 400 ng of total RNA was purified and used as a template for cDNA synthesis by reverse transcriptase with random primers. The specificity of the strand was obtained by replacing the deoxythymidine triphosphate (dTTP) with the deoxyuridine triphosphate (dUTP). cDNA was subsequently converted to double-stranded DNA that was end-repaired. Ligation of adaptors was followed by a purification step with AxyPrep Mag PCR Clean-up kit (Axygen, Big Flats, NY, USA) by an excision of the strands containing the dUTPs and, finally, by a PCR enrichment step of nine cycles to incorporate specific indexed adapters for the multiplexing. With a DNA screentape D1000 on a TapeStation 2200, the quality of final amplified libraries was examined, and the quantification was done on the QuBit 3.0 fluorometer (Thermo Fisher Scientific, Canada). Subsequently, mRNA-seq libraries with unique index were pooled together in equimolar ratio and sequenced for paired-end 100 bp sequencing on NovaSeq 6000 flowcell S1 at the Next-Generation Sequencing Platform, Genomics Center, CHU de Québec-Université Laval Research Center, Quebec City, Canada. The average insert size for the libraries was 290 bp, and the mean coverage/sample was 24M paired-end reads.

### Transcriptome analysis

Reads were trimmed using fastp V0.20.0. To ensure the quality of the reads, a quality check was performed on raw and trimmed data using FastQC v0.11.8 and MultiQC v1.8. The quantification was performed with Kallisto v0.46.2 against the Macaca mulata transcriptome (Mmul_10 downloaded from Ensemble release 103). The main component analysis was completed using the FactoMineR v2.4 R package. The PCA and volcano graphical representations were realized using the ggplot2 v3.3.3 package. Differential expression analysis was also realized using the DESeq v1.30.1 package. This package overcomes the lack of power due to the high uncertainty of the variance estimation by pooling information across genes (108). It was developed to work robustly with few replicates for each condition. Differentially expressed genes were defined as genes with an adjusted *P*-value smaller or equal to 0.05, a fold change greater than 2.5 or smaller than 0.4, and a mean TPM count across all samples greater or equal to 2. All R analysis were done with R v4.0.3. Using the ToppGene (ToppFun) webtool, Gene Ontology Biological Process terms were analyzed by functional enrichment analysis (109). Gene classifications in Table S4 were performed by combining three web interfaces: g g:Profiler (110), GeneCodis 4.0 (111), and ToppGene (109). Using the Reactome and KEGG Knowledgebase interface, genes related to metabolism pathways were classified into categories (112, 113). Gene-set clustering analysis was constructed using ShinyGO service v. 0.75 (114).

### Statistical analysis

Statistics were performed using GraphPad Prism 9 software. The non-parametric Mann-Whitney test and the Wilcoxon test were used for comparison. *P*-values <0.05 indicate a significant difference. The Spearman analysis was used for correlations.

## Data Availability

All data are available in the main text or the supplemental materials. Data set of TPM counts from transcriptomic analysis is also permanently accessible from 10.5281/zenodo.7843666.

## SUPPLEMENTAL MATERIAL

Supplemental figures spectrum.01687-23-s0001.pdf Figures S1 to S3.

Supplemental tables spectrum.01687-23-s0002.pdf Tables S1 to S5.

ASM does not own the copyrights to Supplemental Material that may be linked to, or accessed through, an article. The authors have granted ASM a non-exclusive, world-wide license to publish the Supplemental Material files. Please contact the corresponding author directly for reuse.
