# Supplementary material for: Lipid metabolic reprogramming of hepatic CD4+ T cells during SIV infection
Source: Microbiol Spectr. 2023 Sep 1;11(5):e01687-23. doi: 10.1128/spectrum.01687-23 (PMC10581067; doi:10.1128/spectrum.01687-23)
Supplement: Supplemental figures — Figures S1 to S3. [file spectrum.01687-23-s0001.pdf]

Supplementary Figures

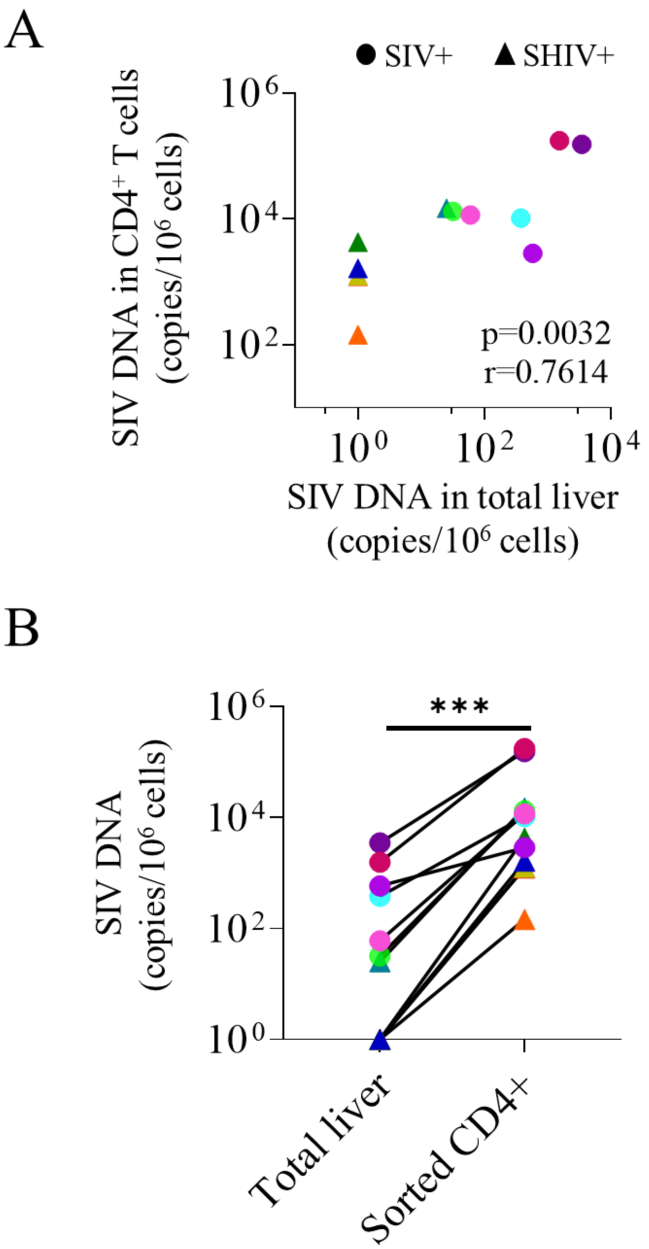

Supplementary Fig. 1. Cell associated-viral DNA in total liver and sorted liver CD4$^+$ T cells. **(**A) Correlation between frequencies of viral DNA in total liver and frequencies of viral DNA in sorted hepatic CD4$^+$ T cells. Spearman analysis was used for correlations. The r and p values are indicated in the figures. (B) Levels of cell-associated viral DNA in total liver in comparison to sorted hepatic CD4$^+$ T cells. Each colored symbol represents one individual (n=12). Cell-associated viral DNA are expressed as copies per 10^6 cells. A Wilcoxon test was performed, *** indicates $p<0.001$.

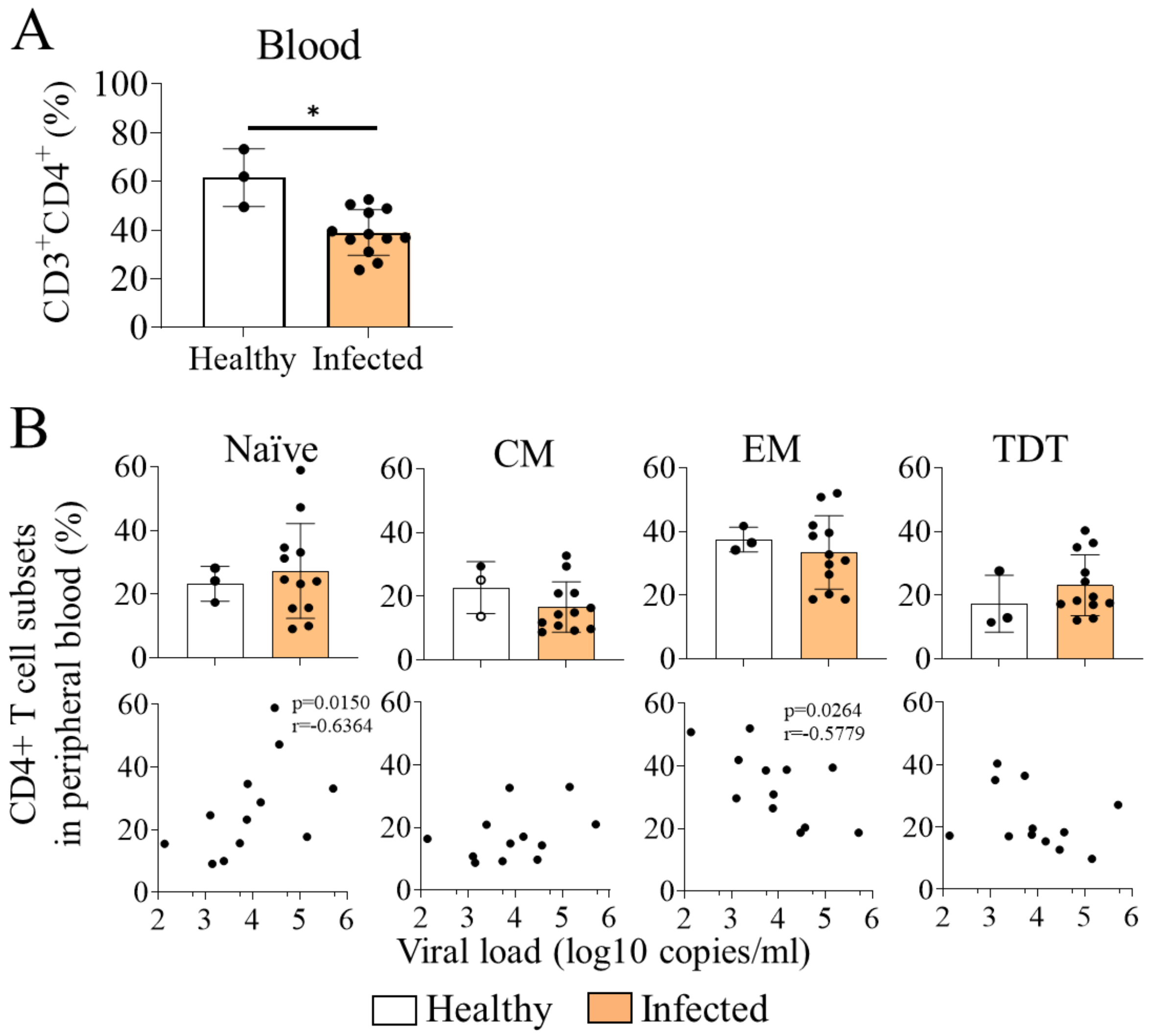

Supplementary Fig. 2. CD4$^+$ T cell dynamic in the blood of healthy uninfected and SIV/SHIV-infected RMs. (A) Histograms show the percentages of CD3$^+$CD4$^+$ T cells of healthy uninfected and SIV/SHIV-infected RMs. (B) Histograms (top panel) show the percentages of naïve (CD62L$^+$CD45RA$^+$), central memory (CM, CD62L$^+$CD45RA$^-$), effector memory (EM, CD62L$^-$CD45RA$^-$) and terminal differentiated (TDT, CD62L$^-$CD45RA$^+$) blood CD4$^+$ T cells of healthy uninfected (white) and SIV/SHIV-infected (orange) RMs. Correlations (bottom panel) between viral load and percentages of blood CD4$^+$ T cell subsets of infected individuals. * indicates $p<0.05$. Spearman analysis was used for correlations. The r and p values are indicated in the figures.

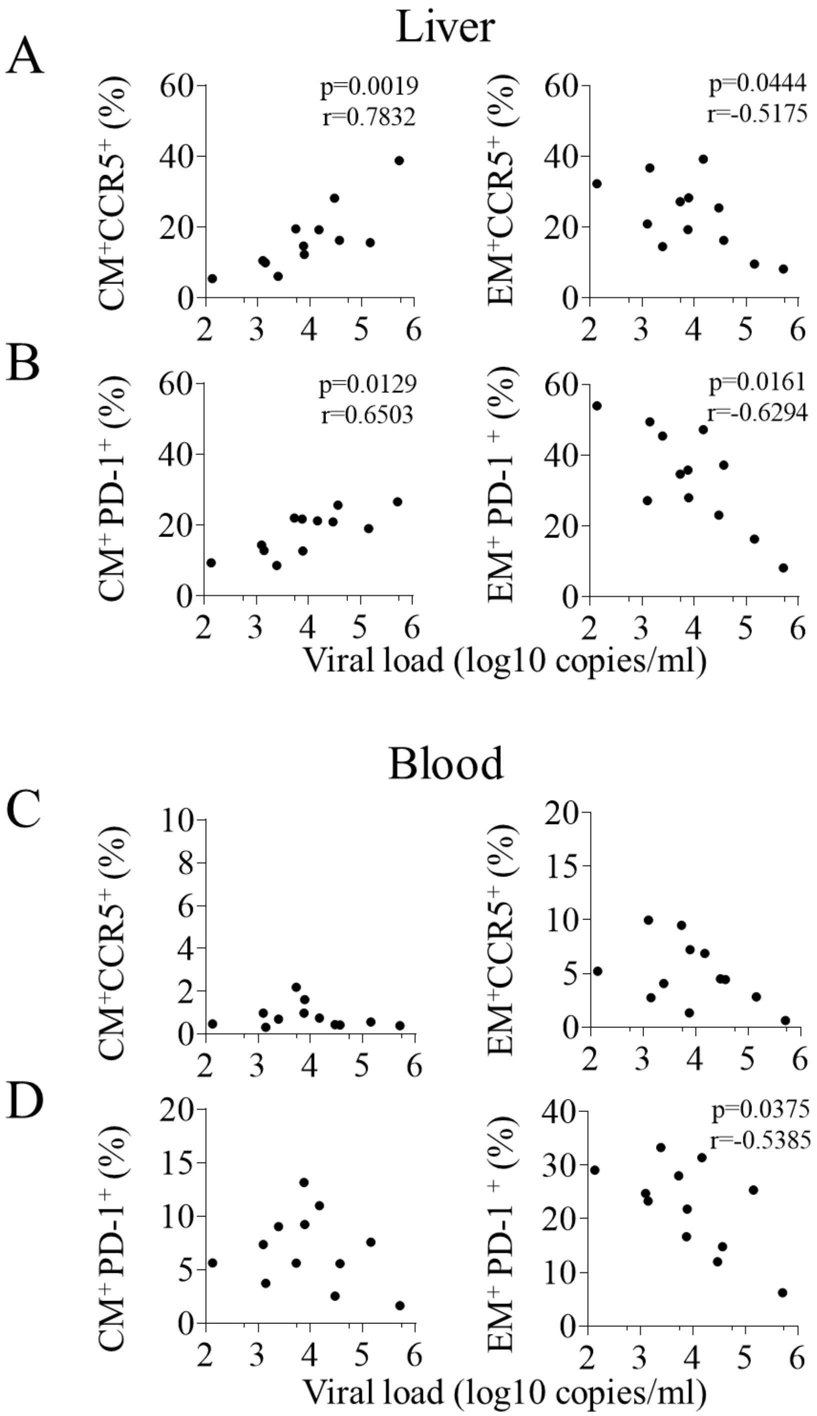

Supplementary Fig. 3. Correlation between hepatic CD4$^+$ T cell subsets expressing CCR5/PD-1 and viral load. (A-B) Correlations between liver CD4$^+$ T cell subsets expressing (A) CCR5 and (B) PD-1 and viral load. (C-D) Correlations between blood CD4$^+$ T cell subsets expressing (C) CCR5 and (D) PD-1 and viral load. Spearman analysis was used for correlations. The r and p values are indicated in the figures.
