# Supplementary material for: Lipid metabolic reprogramming of hepatic CD4+ T cells during SIV infection
Source: Microbiol Spectr. 2023 Sep 1;11(5):e01687-23. doi: 10.1128/spectrum.01687-23 (PMC10581067; doi:10.1128/spectrum.01687-23)
Supplement: Supplemental tables — Tables S1 to S5. [file spectrum.01687-23-s0002.pdf]

Supplementary Tables

	Macaque's symbol	Day of sacrifice post-infection	Viral load	$CD4^+$ T cells (counts/mm^3)	CD4/CD8 Ratio
SIV-	■	0	0.00E+00	ND	1.0
	■	0	0.00E+00	ND	2.7
	■	0	0.00E+00	ND	1.6
SHIV+	▲	30	1.26E+03	1423	0.6
	▲	60	1.49E+04	699	0.6
	▲	120	1.35E+02	758	1.0
	▲	120	1.40E+03	2143	1.0
	▲	120	7.81E+03	719	0.6
	▲	120	7.57E+03	1255	0.7
SIV+	●	295	3.68E+04	602	0.4
	●	307	5.14E+05	190	0.3
	●	314	6.54E+03	533	0.2
	●	315	2.95E+04	415	0.4
	●	331	1.43E+05	684	0.9
	●	335	2.45E+03	1535	1.1

Table S1. Virological and immunological parameters of rhesus macaques included in our study. CD4/CD8 ratio was calculated from the percentages of T cells determined by flow cytometry. ND: Not Done.

Cluster of differentiation	Fluorochrome	Clone	Manufacturer
CD3	Alexa700	SP34-2	BD Biosciences
CD4	V500 / APC	L200	BD Biosciences
CD20	APC Cy7	2H7	BD Biosciences
TCR Vα24-Jα18 (NKT)	BV510	6B11	BioLegend
CD62L	FITC	SK11	BD Biosciences
CD45RA	EDC	2H4LDH11LDB9	Beckman Coulter
PD-1	BV421	EH12.2H7	BioLegend
CD195 (CCR5)	BV786	3A9	BD Biosciences

Table S2. Antibody used.

	Primers and probes	Nucleotides sequences 5'-3'
SIV/SHIV	SIV/SHIV Gag-F	GCA GAG GAG GAA ATT ACC CAG TAC
	SIV/SHIV Gag-R	CAA TTT TA CCC AGG CAT TTA ATG TT
	SIV/SHIV Gag-Probe	6FAM TGT CCA CCT GCC ATT AAG CCC GA TAMRA
	SIV DNA Nef-For	GGC CCT ACA GAG AAT TCG AGA
	SIV DNA Nef-Rev	ACT GAT ACC CCT ACC AAG TCA
	Nested SIV DNA-For	TCC CTA GGA GGA TTA GAC AAG G
	Nested SIV DNA-Rev	CTC TCT TCA GCT GGG TTT CTC
	SIV DNA-Probe	56FAM AGC TCA CTC ZEN TCT TGT GAG GGA CAG A 3IABkFQ
	SHIV DNA Nef-For	GGG GAG ACT TAT GGG AAA CT
	SHIV DNA Nef-Rev	ATA AAT CCC TTC CAG TCC CC
	Nested SHIV DNA-For	GGA GAA ACC CAG CTG AAG A
	Nested SHIV DNA-Rev	CAT GTC TAT TGC CAA TTT GTA ACT C
	SHIV DNA-Probe	56FAM TGT CCC TGA ZEN TTG TAT TTC TGT CCC TCA C 3IABkFQ
Macaque rhesus	ADN18S-For	CCT CCA ATG GAT CCT CGT TA
	ADN18S-Rev	AAA CGG CTA CCA CAT CCA AG

Table S3. Primers and probes used.

Immune response (GO:0006955, p=2.810E-22)			Regulation of cell death (GO:0010942, p=5.973E-7)			Cellular metabolic process (GO:0008152, p=1.908E-34)			Transport (GO:0006810, p=4.502E-32)		Organelle organization (GO:0006996, p=8.727E-21)
**IFN**	**Cell surface receptor**	**Inflammatory molecules**	**Apoptosis**	**Ferroptosis**	**Autophagy**	**Lipid**	**Carbohydrate**	**Mitochrondria**			
DDX56	ADAM15	ATF5	TNFSF12	AIFM2	AKT1	ABHD11	CSGALNACT2	ACADVL	ADGRE2	RP2	ARHGDIA
DDX58	CD38	C1QB	PSMF1	AKR1C1	VPS51	ABHD4	EXT2	ACSF2	ANO10	SH3GL1	CDCA8
IFI27	CD5L	C1QBP	BAD	IREB2	CHMP2B	AKR1C1	GYG1	ATP13A1	APBA3	SIL1	CEP250
IFI44	CD7	CFD	PSMB2	HMOX1	RETREG1	AKR7A2	PYGM	CHCHD3	APOO	SLC22A18	CFAP298
IFI44L	CD70	CFP	CDC37	SLC3A2	LGALS8	ALDH16A1	SDHC	DDX28	ARFGAP3	SLC24A1	CORO6
IFI6	CRTAM	CTSB	PSMD3	ACSL5	DAPK3	B3GALT4		DNAJA3	ATL2	SLC25A24	CSRP2
IRF7	CSF1R	CTSH	BCL3		GPR137B	CERS6		ELAC2	ATP1B1	SLC35A2	DCTN6
IRF8	CX3CR1	CTSL			AIMP2	CREM		ERAL1	ATP2C1	SLC39A1	FAM110A
MX1	CXCR3	CYSTM1			EI24	DGKZ		FAHD1	BBIP1	SLC3A2	FOXRED1
MX2	IL21R	ETS2			MAP1LC3A	ESRRA		FARS2	BEST1	SLC4A2	FSCN1
SOCS1	IL4R	ETV3			GABARAPL1	GPAT4		ME2	BORCS5	SLC66A2	GORASP2
TRIM5	KLRD1	ETV7			SDCBP	GSTM5		MRPL28	CEP120	SNX11	GPR65
	LILRA3	FOSL2			MCOLN1	HMGCR		MRPL52	CLTB	SNX8	KIZ
	MAMU-DMB	GTF3C1			TBC1D17	ILVBL		MRPS6	DYNC2I2	STX10	KLHDC8B
	MAMU-DOB	GTF3C5				ITPKC		MTCH1	ESYT2	STX18	MAD1L1
	MAMU-DRB1	GZMA				KAT5		POLDIP2	FCHO1	STX2	MAD2L1BP
	PDCD1	IFRD2				LDLR		RARS2	GOLPH3L	SUN1	MAU2
	SLAMF7	IL7				LGMN		RPUSD3	JAKMIP1	TACC3	NCAPH2
		KLF2				MLST8			KCNIP2	TFG	PARVG
		LAT2				MTMR14			LDLRAP1	TIMM29	PDLIM5
		LYZ				NR1H3			LSG1	TM9SF4	PHACTR1
		MAFF				NUDT7			LTV1	TMED1	PLEKHG2
		MEF2D				PEMT			MAIP1	TOM1L2	RMDN1
		NFIC				PGAP2			MAMDC4	TTYH2	SCO1
		NFKBIE				PIGH			MFSD3	XKR8	STMN3
		NPDC1				PIP4P1			MFSD6	XPO7	TAGLN
		PIAS4				PITPNM2			MKLN1		TCTN3
		RAP1GAP2				PLCB2			MPC1		TNNT3
		RNF4				PLCD1			NUCB1		TOGARAM1
		S100A9				PLEK			OCIAD1		TUBB2B
		SIRT2				PNPLA7			PRG4		UQCC1
		STAT5A				PRKD3			RAB11FIP5		WRAP73
		TFE3				SERINC5			RAB31		FLNA
		TGFBI				SPTLC1			RAB35		
		TGIF2				STAR			RAE1		
		THAP3				THRA			RFTN1		
		ZNF267				TNFAIP8L2			RHOBTB3		
		ZNF32				TSPO			RIN3		

Table S4. Gene classification of upregulated expressed gene

**Interferon response**	**log2FoldChange**	**pvalue**
DDX58	7.37	3.12E-05
MX2	6.64	4.90E-05
MX1	5.44	7.40E-05
TRIM5	7.68	4.33E-04
IFI6	5.52	4.63E-04
IRF8	5.08	2.35E-03
IFI27	5.77	3.91E-03
RAE1	4.74	8.15E-03
IFI44	4.13	8.63E-03
IRF7	4.62	1.23E-02

**Inflammatory molecules**	**log2FoldChange**	**pvalue**
RAP1GAP2	8.94	3.10E-07
C1QB	8.89	6.37E-07
LYZ	9.64	8.26E-07
GZMA	9.44	2.96E-06
CFD	9.75	5.15E-06
CTSL	8.18	6.43E-06
LAT2	8.60	1.43E-05
NPDC1	7.07	5.31E-05
IL7	7.72	9.60E-05
CYSTM1	7.72	1.85E-04

**Cell death regulation**	**log2FoldChange**	**pvalue**
DAPK3	7.54	7.63E-07
GPR137B	7.50	1.26E-05
PSMD3	8.23	1.91E-05
BCL3	7.27	3.21E-05
GABARAPL1	7.16	5.61E-05
PSMF1	6.09	9.04E-05
AIMP2	7.63	9.67E-05
EI24	6.93	9.70E-05
MAP1LC3A	7.42	2.07E-04
TNFSF12	5.54	2.46E-04

**Metabolism**	**log2FoldChange**	**pvalue**
LGMN	9.31	1.43E-06
PNPLA7	7.91	1.43E-06
STAR	8.62	9.53E-06
ABHD4	8.54	1.93E-05
PLCD1	8.16	3.71E-05
PITPNM2	7.95	5.03E-05
NR1H3	7.75	7.82E-05
EXT2	5.41	4.31E-04
CERS6	7.13	5.07E-04
PEMT	5.74	5.84E-04

**Cell surface receptors**	**log2FoldChange**	**pvalue**
CD5L	9.14	2.09E-05
CSF1R	8.91	2.43E-05
ADAM15	7.83	4.58E-05
CX3CR1	6.91	1.65E-04
CXCR3	8.36	2.71E-04
KLRD1	6.89	2.99E-04
CRTAM	6.43	3.45E-04
IL21R	7.11	4.73E-04
LILRA3	7.01	5.54E-04
CD38	5.20	5.69E-04

**Gene regulation**	**log2FoldChange**	**pvalue**
FOSL2	7.63	1.13E-05
ETV7	8.48	3.82E-05
TFE3	6.85	7.82E-05
RNF4	7.37	1.97E-04
IFRD2	6.68	2.32E-04
KLF2	6.66	2.74E-04
ATF5	5.42	4.17E-04
ZNF32	7.02	6.45E-04
PIAS4	5.74	6.94E-04
ETV3	4.70	1.68E-03

**Mitochrondria**	**log2FoldChange**	**pvalue**
ND4L	-17.93	1.07E-19
ND4	-7.12	1.67E-06
RPUSD3	8.75	5.91E-06
POLDIP2	8.47	3.06E-05
ACADVL	6.62	3.21E-05
MTCH1	6.52	9.56E-05
ELAC2	6.08	1.24E-04
CHCHD3	6.10	1.22E-03
ME2	6.19	2.65E-03
ERAL1	4.67	3.44E-03

Table S5. Top 10 of differentially expressed genes in selected pathways.
